# Effects of Annealing Temperature on the Oxygen Evolution Reaction Activity of Copper–Cobalt Oxide Nanosheets

**DOI:** 10.3390/nano11030657

**Published:** 2021-03-08

**Authors:** Geul Han Kim, Yoo Sei Park, Juchan Yang, Myeong Je Jang, Jaehoon Jeong, Ji-Hoon Lee, Han-Saem Park, Yong Ho Park, Sung Mook Choi, Jooyoung Lee

**Affiliations:** 1 Department of Energy & Electronic Materials, Surface Materials Division, Korea Institute of Materials Science, Changwon 642831, Korea; geulhan0726@kims.re.kr (G.H.K.); qkrdbtp@kims.re.kr (Y.S.P.); juchana83@kims.re.kr (J.Y.); propojang@kims.re.kr (M.J.J.); reliability@kims.re.kr (J.J.); jhlee@kims.re.kr (J.-H.L.); 2 Department of Materials Science and Engineering, Pusan National University, Busan 46241, Korea; 3 IT&New Application Battery Development, LG Chem, Daejeon 34112, Korea; hanseam79@gmail.com

**Keywords:** oxygen evolution reaction, transition metal oxide catalyst, water splitting, electrode materials, non-noble-metal catalysis, electrodeposition

## Abstract

Developing high performance, highly stable, and low-cost electrodes for the oxygen evolution reaction (OER) is challenging in water electrolysis technology. However, Ir- and Ru-based OER catalysts with high OER efficiency are difficult to commercialize as precious metal-based catalysts. Therefore, the study of OER catalysts, which are replaced by non-precious metals and have high activity and stability, are necessary. In this study, a copper–cobalt oxide nanosheet (CCO) electrode was synthesized by the electrodeposition of copper–cobalt hydroxide (CCOH) on Ni foam followed by annealing. The CCOH was annealed at various temperatures, and the structure changed to that of CCO at temperatures above 250 °C. In addition, it was observed that the nanosheets agglomerated when annealed at 300 °C. The CCO electrode annealed at 250 °C had a high surface area and efficient electron conduction pathways as a result of the direct growth on the Ni foam. Thus, the prepared CCO electrode exhibited enhanced OER activity (1.6 V at 261 mA/cm^2^) compared to those of CCOH (1.6 V at 144 mA/cm^2^), Co_3_O_4_ (1.6 V at 39 mA/cm^2^), and commercial IrO_2_ (1.6 V at 14 mA/cm^2^) electrodes. The optimized catalyst also showed high activity and stability under high pH conditions, demonstrating its potential as a low cost, highly efficient OER electrode material.

## 1. Introduction

Electrochemical water splitting is an effective method of hydrogen production [1,2]. In addition, it can be ecofriendly because of the possibility of using renewable energy sources such as photovoltaic, wind, and hydroelectric power. The theoretical potential required for water splitting is 1.23 V [3,4]. However, an additional overpotential is required in both the oxygen and hydrogen evolution reactions (OER and HER, respectively). Specifically, the kinetics of OER is slow and require a higher overpotential than HER at actual operating current density of electrochemical water splitting [5]. In this regard, developing an electrocatalyst that can expedite the OER has drawn much interest and intense efforts [6,7]. The commonly used Ir- or Ru-based electrodes have high OER activities but are costly because of the use of noble metals, which have limited reserves [8,9]. Therefore, there has been ongoing research into earth-abundant transition metal-based electrodes that can replace noble metal electrodes. For example, OER catalysts comprising inexpensive and abundant resources such as transition metal (such as Co, Ni, Fe, and Cu) oxides [10,11], phosphides [12,13], and borates are drawing attention [14,15]. In particular, Co_3_O_4_ has emerged as an attractive candidate because of its efficient electrode performance resulting from its nanostructure and low cost [16,17]. However, the electrical conductivity of Co is low; thus, investigation into methods to improve the conductivity is required [18,19]. Methods to improve electrical conductivity and activity for electrocatalysts include composite transition metal catalyst synthesis, structure optimization and surface area enhancement using highly conductive materials [20,21]. Previous studies were conducted on Cu*_x_*Co_2-*x*_O_4_ (CCO) catalysts, which have higher conductivities as a result of the Cu doping of Co_3_O_4_. In addition, various approaches such as chemical deposition, hydrothermal methods, and electrodeposition have been used to improve the surface morphology and electrochemical and crystallographic properties of the CCO catalyst [22,23].

Alkaline water electrolysis (AWE) is a mature technology for H_2_ production and is the most used water electrolysis method [24,25]. This AWE cells are powered by a highly concentrated aqueous alkaline electrolyte over 20 wt.% KOH of NaOH (3.6 M KOH or NaOH). [26]. However, it is difficult to secure electrode stability with high concentration alkaline electrolyte. Therefore, many AWE cells currently use steel or nickel alloy plated steel. Despite the recent results of many OER catalyst studies, there are not many reported studies on OER evaluation and stable OER catalysts above 20 wt.% KOH other than a few papers [27,28,29]. Therefore, development of stable electrodes even above 20 wt.% KOH is strongly needed. Thus, research is needed to develop a practical and convenient method for optimizing catalysts with a variety of structures and excellent electrochemical performance. Catalyst optimization can improve stability and performance, as well as electrical conductivity. For optimization, the temperature, pressure, and reactants can be controlled as parameters. However, optimization via temperature-controlled heat treatment is the easiest method to improve catalyst stability, as well as change the catalyst structure; furthermore, it is an established process.

**Figure 1:**
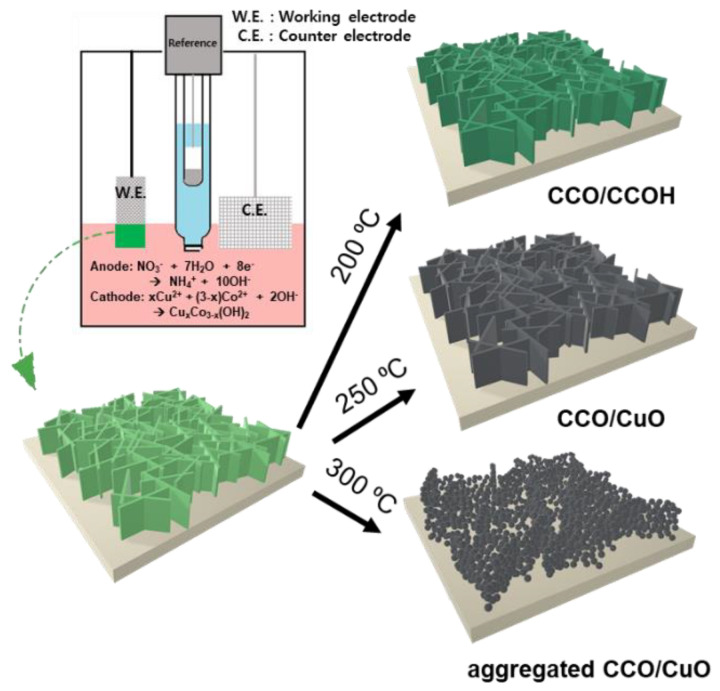
Schematic illustration of CCO catalyst preparation using electrodeposition and heat treatment at 200, 250, and 300 °C.

In this study, the CCO electrode was synthesized by electrodepositing copper-cobalt hydroxide (CCOH) on nickel foam and then heat treatment, and catalytic activity and durability of the electrode were optimized. In particular, we found that the nanosheets turned into particles on the surface after heat treatment at 300 °C or higher (Figure 1). When comparing the electrochemical properties of the electrodes at a current density of 20 mA/cm^2^, the lowest overpotential of 276 mV was achieved for the electrode annealed at 250 °C. Furthermore, the catalytic performance was tested at a high pH, and it remained stable for 100 h.

## 2. Materials and Methods

### 2.1. Preparation of Copper–Cobalt Oxide Electrodes by Cathodic Electrodeposition

To surface oxidation (NiO) of the Ni foam (0.7 cm × 0.7 cm) substrate, etching with HCl (5 M) for 30 min was carried out, and the residual acid was removed from the substrate by washing with deionized (DI) water [30,31]. To prepare the electrodeposition solution, copper (II) nitrate hemi(pentahydrate) (10 mM, Cu(NO_3_)_2_·2.5H_2_O, ≥98%, Sigma-Aldrich Co., Ltd., St. Louis, MO, USA) and cobalt (II) nitrate hexahydrate (50 mM, Co(NO_3_)_2_·6H_2_O, ≥98%, Sigma-Aldrich Co., Ltd., St. Louis, MO, USA) were prepared in 500 mL of DI water and stirred for 30 min. The Ni foam substrate with surface oxide removed, a Pt mesh (3 cm × 4 cm), and a saturated calomel electrode (SCE) were used as the working (cathode), counter (anode), and reference electrodes, respectively (the distance of each electrode is 1 cm). Then, a constant potential of −1 V vs. SCE was applied for 5 min at 30 °C. After this process, the color of the nickel foam turned dark green. The electrode made by electrodeposition was washed with prepared DI water, and the dried at room temperature. Subsequently, heat treatment (annealing) was performed on the electrodeposited catalysts by heating at a rate of 1 °C/min to 150, 200, 250, and 300 °C in air for 3 h. The catalysts are labeled as CCO-*x* °C.

### 2.2. Physicochemical Characterization 

Field-emission scanning electron microscopy (FE-SEM, JEOL, JSM-7001F, Tokyo, Japan) were used to observe the surface morphologies of the CCO electrodes. Transmission electron microscopy (TEM, Thermo Fisher Scientific, Talos F200X, Waltham, MA, USA) were used to observe the morphology and structure of CCO-250 °C nanosheets. Crystallinity analysis of copper-cobalt oxide was performed using X-ray diffractometry (XRD, RIGAKU, D/MAX-2500V, Rigaku Corporation, Akishima, Japan), which was performed using copper radiation (1.54 Å, Cu Kα) generated at a voltage and current of 40 kV and 250 mA, respectively. Measurements were carried out at a scan rate of 1°/min from 10° to 70° in 2*θ*. In addition, the chemical state of the electrode before and after testing the stability in 1 M KOH was obtained using X-ray photoelectron spectroscopy (XPS, Thermo Fisher, ESCALAB 250, Waltham, MA, USA) using an Al Kα (1486.6 eV) radiation source.

### 2.3. Electrochemical Characterization

Linear sweep voltammetry (LSV) and cyclic voltammetry (CV) were performed using a potentiostat (VMP3, Bio-Logic, Seyssinet-Pariset, France) for OER measurements. The measurements were conducted under alkaline conditions in a three-electrode system, and Pt mesh and Hg/HgO (1 M KOH) were used as the counter and reference electrodes, respectively. The OER tests were conducted in 1 M KOH solution at room temperature. Ni foam with an area of 0.49 cm^2^ was used as the working electrode. All potentials were corrected according to *V*_RHE_ = *V*_Hg/HgO_ + 0.059pH + 0.098 V, where *V*_RHE_ is the potential versus that of the reversible hydrogen potential, *V*_Hg/HgO_ is the potential versus that of the Hg/HgO electrode, and pH is the electrolyte pH. All electrochemical results were 85% *IR*-corrected [32]. A stability test was performed by applying a constant current of 20 mA/cm^2^ for about 100 h. The Tafel slope was calculated using Equation (1).

(1) $$\eta =A\times \log_{10}\left(\frac{I}{I_{0}}\right)$$

here, *η* represents the overpotential (mV), *I* is the current density (mA/cm^2^), *I*_0_ is the exchange current density (mA/cm^2^), and *A* is the Tafel slope (mV/dec). Thus, the OER mechanism of the electrode was compared. For electrochemical impedance spectroscopy (EIS), measurements were conducted in 1 M KOH in a frequency range of 100 mHz to 200 kHz at a potential of 1.34 V. The electrochemical double layer capacitance (*C*_dl_) was obtained using CV measurements in the non-Faradaic region at a scan rate of 20 to 100 mV/s).

## 3. Results

**Figure 2:**
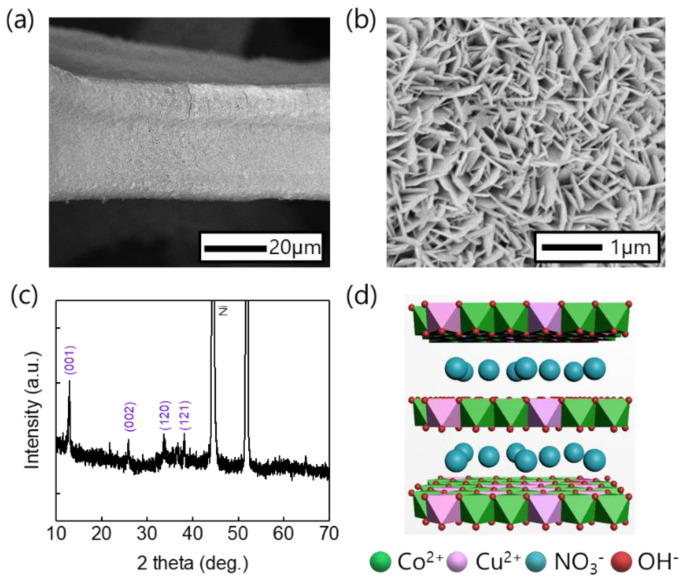
(**a**,**b**) SEM images and (**c**) XRD pattern of electrodeposited CCOH. (**d**) Proposed structure of Cu_1-x_Co_x_(OH)_2_(NO_3_).

**Figure 3:**
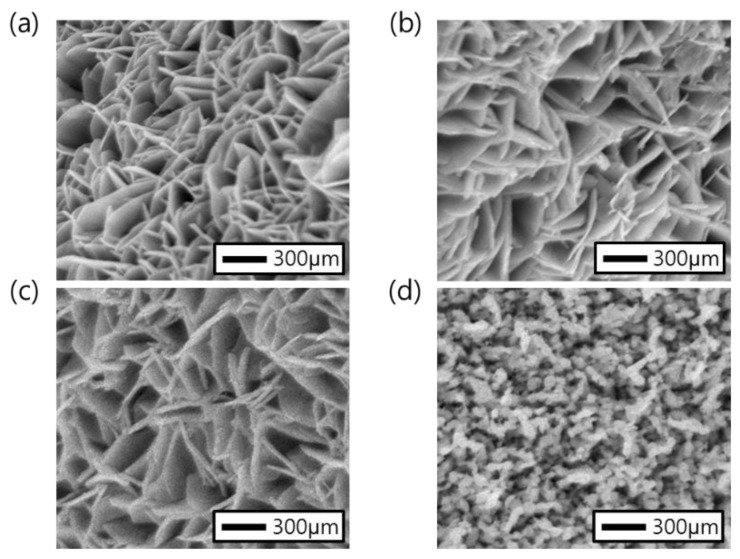
SEM images showing the changes in CCO morphology with temperature: (**a**) 150, (**b**) 200, (**c**) 250, and (**d**) 300 °C, respectively.

**Figure 4:**
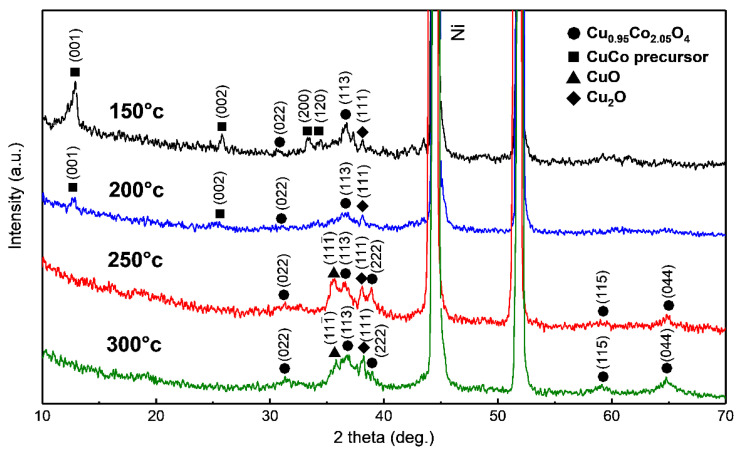
XRD patterns of CCO after treatment at different temperatures: (from top to bottom) 150, 200, 250, and 300 °C.

CuCo precursors were synthesized on Ni foam substrates from which surface Ni oxide was removed using electrodeposition using previously reported methods. FE-SEM analysis confirmed that the CuCo precursor had been uniformly deposited on the surface of the Ni foam in a nanosheet structure (Figure 2a,b). The XRD patterns (Figure 2c) of the CuCo precursor electrodes treated at all temperatures contain diffractions at 2*θ* = 12.9°, 25.8°, 33.6° and 38.1° which correspond to the (001), (002), (120) and (121) planes of Cu_1__−__x_Co_x_ (OH)_2_(NO_3_) (CCOH), respectively [33,34]. After heat treatment, changes in the morphology and structure were observed via SEM and XRD analyses, respectively. The SEM images of the electrodes annealed at temperatures below 250 °C show that the surface morphology retained a nanosheet structure (Figure 3a–c). However, after annealing at 300 °C or above, the surface morphology became particle-like because of the structural breaking and aggregation of the nanosheets (Figure 3d). The XRD patterns (Figure 4) of the electrodes treated at all temperatures contain diffractions at 2*θ* = 31.2°, 36.8°, and 65.1°, which correspond to the (022), (113), and (044) planes, respectively, of Cu_0.92_Co_2.08_O_4_ (ICSD card No. 98-003-6356) with an inverse spinel structure. The minor peaks at 35.5° and 38.8° were indexed to the (002) and (111) planes, respectively, which were identified as CuO (ICSD card No. 98-009-2365). In addition, for the electrodes treated at 150 and 200 °C, diffractions at 2*θ* = 13.0° and 25.7° were observed, and these correspond to CCOH. With increase in the heat treatment temperature, the nitrate (NO_3_^−^) contained in CCOH disappeared, the intensity of the CCO peak increased, and only diffractions corresponding to oxide species were observed.

**Figure 5:**
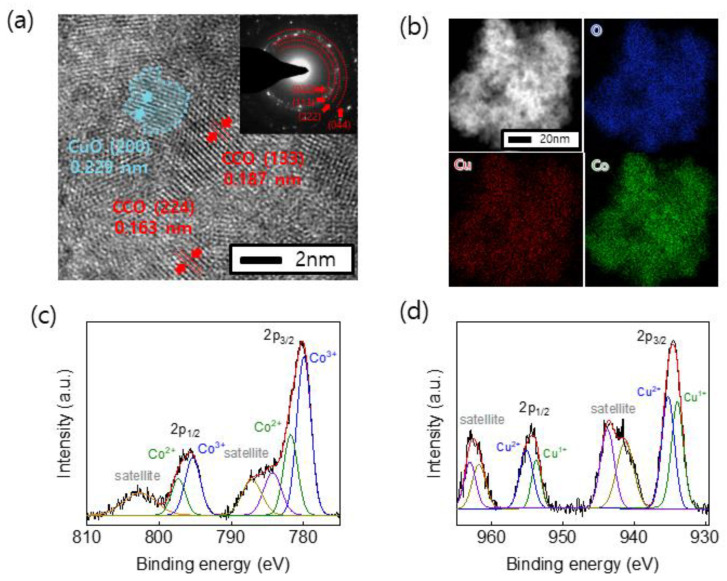
(**a**) High-resolution TEM image (blue, CuO *d*_(200)_ = 0.229 nm; red, CCO *d*_(133)_ = 0.187 nm and *d*_(224)_ = 0.163 nm), (**b**) EDS elemental maps of (clockwise) O, Co, and Cu, and (**c**) Co 2p and (**d**) Cu 2p XPS core level spectrum of CCO-250 °C.

The Co oxide structure is more stable than Co hydroxide for OER [35,36]. Thus, TEM and XPS analyses were performed to enable the precise structural analysis and chemical states of oxide species in the electrodes annealed at 250 °C, respectively. These nanosheets of CCO-250 °C were detached from the electrode by sonication, and then characterized by TEM. We confirmed that CuO and CCO were alloyed on the Ni foam substrate (Figure 5a). In addition, the components of the CCO-250 °C electrode were analyzed using energy-dispersive X-ray spectroscopy (EDS, Figure 5b). As a result of the analysis, the uniform distribution of Cu, Co, and O atoms in the CCO-250 C electrode was confirmed. When examining the Co 2p spectrum, Co^2+^ peaks were observed at 797.4 and 781.9 eV, and Co^3+^ peaks were observed at 795.4 and 779.9 eV (Figure 5c). The Cu 2p spectrum also contained Cu^+^ ion peaks at 934.0 and 953.8 eV and Cu^2+^ ion peaks at 935.2 and 955.2 eV (Figure 5d). The peak quantifications for the elements Co^2+^, Co^3+^, Cu^+^ and Cu^2+^ presented in CCO-250 °C were 34, 17, 23, and 26%, respectively. In general, in the spinel structure of Cu_x_Co_3-x_O_4_, when x is 0.7 or more, Co^3+^ enters the tetrahedral site of spinel structure and forms an inverse spinel structure. These results indicated the formation of Cu_0.92_Co_2.08_O_4_, CuO, and Cu_2_O through the electrodeposition and heat treatment [37].

**Figure 6:**
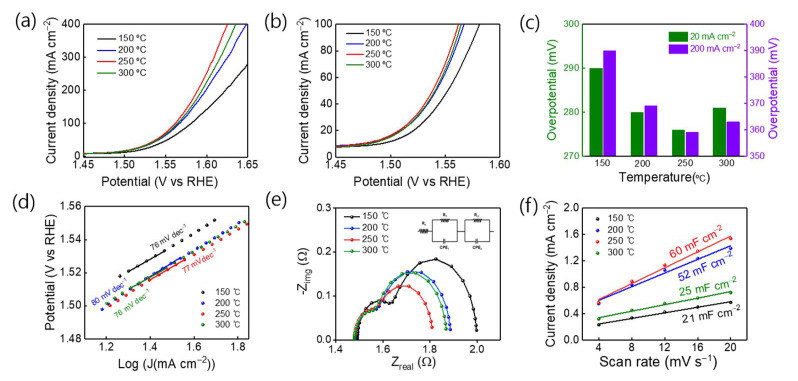
(**a**,**b**) LSV curves (85% *IR*-compensated) and (**c**) overpotentials at various current densities for CCO catalysts (150, 200, 250, and 300 °C) in 1 M KOH at room temperature. (**d**) Tafel plots, (**e**) EIS results, and (**f**) *C*_dl_ of CCO catalysts (150, 200, 250, and 300 °C).

In addition, we investigated the electrocatalytic properties of the samples for the OER with respect to the heat treatment temperature. Figure 6a shows the polarization curves on the reversible hydrogen electrode (RHE) scale recorded at a scan rate of 2 mV/s in 1 M KOH solution. As the heat treatment temperature increased from 150 to 200 to 250 °C, at a low current density of 20 mA/cm^2^, the overpotentials decreased from 291 to 280 to 276 mV, respectively. In contrast, that of the electrode annealed at 300 °C increased to 281 mV. The same trend was observed at a high current density of 200 mA/cm^2^, and the lowest overpotential of 359 mV was obtained after heat treatment at 250 °C (Figure 6b). The performance of the proposed OER electrodes was compared at a current density of 10 mA/cm^2^ (Figure 6c). The measured overpotential of the CCO-250 °C electrode was lower than that of the reported OER catalysts under the same or similar conditions (Table 1) [38,39,40,41,42,43,44,45,46]. This result indicates that the CCO-250 °C electrode has higher OER catalytic activity compared to other catalysts. The Tafel slope relates the rate of the electrochemical reaction and the overpotential.

*η* = *a* + *b* log *j* (2)

here, *η* is the overpotential, *b* is the Tafel slope, and *j* is the current density [47,48]. The Tafel slopes of the CCO electrode were 76, 77, 77, and 75 mV/dec for the electrodes treated at 150, 200, 250, and 300 °C, respectively, at an onset potential of 1.55 V (Figure 6d). These Tafel slope values were not affected by the temperature change and show similar trends, suggesting that even when the heat treatment temperature changes, the same OER mechanism applies and the catalyst maintains the same reaction rate. Electrochemical impedance spectroscopy (EIS) measurements of the CCO electrode were conducted for the samples annealed at 150, 200, 250, and 300 °C at 1.5 V (Figure 6e). The data obtained by impedance analysis is represented as a Nyquist plot, which shows the electrolyte resistance and charge transfer resistance of the electrode. As shown in Figure 6e, two semicircles of the Nyquist plot are observed. The first semicircle in the high frequency region and the second semicircle in the low frequency region represent the solid oxide film resistance (R_1_) and the charge transfer resistance (R_ct_) from the electrolyte to the catalyst surface, respectively [49,50]. The series resistance (*R*_s_) was the same because of the use of the same electrolyte temperature, distance between the electrodes, electrode area, and experimental settings of the cell. The transfer resistance for charge transfer was the lowest for the electrode annealed at 250 °C. In addition, the electrochemically active surface area (ECSA)was determined using the *C*_dl_ to evaluate the catalytic activity of the catalyst. *C*_dl_ was calculated from the CV measurements between −0.05 and 0.05 V versus the open-circuit potential (OCV) at different scan rates. (Figure 6f). The electrodes annealed at 150, 200, 250, and 300 °C have *C*_dl_ values of 21, 52, 60, and 25 mF, respectively, indicating that the highest electrode activity was obtained at 250 °C. At temperatures above 300 °C, the surface area decreased because of particle aggregation, resulting in a decrease in electrode activity. Thus, based on catalyst performance, the electrochemical analysis results confirm that the catalyst performance was optimal at an annealing temperature of 250 °C.

**Figure 7:**
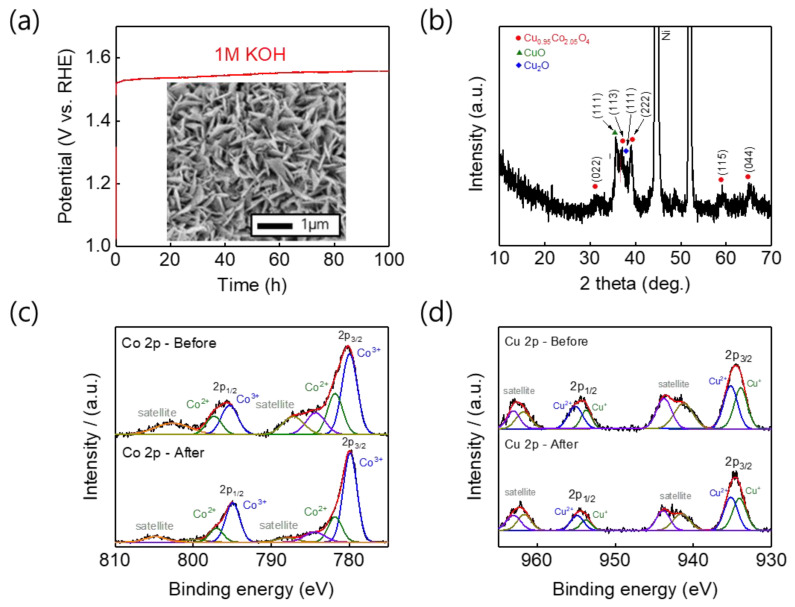
(**a**) Chronopotentiometry curve of CCO-250 °C at a current density of 20 mA cm^−2^ in 1 M KOH for 100 h. Inset show the SEM image of surface morphology for CCO after durability test. (**b**) XRD patterns, (**c**) Co 2p and (**d**) Cu 2p of CCO-250 °C after durability testing.

**Figure 8:**
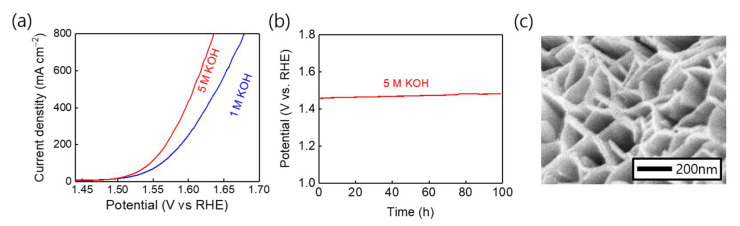
(**a**) LSV curves (85% *IR*-compensated) for CCO-250 °C in 1 and 5 M KOH at room temperature. (**b**) Chronopotentiometry curve of CCO (250 °C) at a current density of 20 mA cm^−2^ in 5 M KOH for 100 h. (**c**) SEM image showing the surface morphology of CCO (250 °C) after durability testing.

Next, stability tests of the CCO-250 °C electrode, which showed the best electrode performance of the prepared electrodes, were conducted. When a constant current at a current density of 20 mA/cm^2^ was applied in a 1 M KOH aqueous solution, the electrode performance was maintained 100 h testing (Figure 7a). An SEM image of the CCO-250 °C catalyst after stability testing is exhibited in Figure 7b, showing that the nanosheet structure of the surface remained intact. After stability testing, XPS analysis was performed. As shown in Figure 7c,d, the Co^2+^ (797.1 and 781.8 eV), Co^3+^ (795.0 and 779.9 eV), Cu^+^ (934.1 and 953.8 eV), and Cu^2+^ (935.2 and 954.9 eV) peak positions remained almost unchanged. The Cu 2p spectrum showed the same Cu^2+^:Cu^+^ ratio as the XPS spectrum before OER. However, the Co^2+^:Co^3+^ ratio in the Co 2p spectrum changed from 67:33 to 81:19. The reason for this difference is that as the stability test proceeded, the Co^2+^ ions were oxidized to Co^3+^ ions, resulting in a change in peak intensity. During alkaline water electrolysis (AWE), the electrode performance and stability must be maintained under extreme conditions. Because AWE systems operate under highly alkaline conditions, the OER activity of the CCO-250 °C electrode was tested in 5 M KOH. By comparing the LSV curves obtained at the same potential but in KOH solutions of different concentrations, we found that the OER catalyst activity was higher in 5 M KOH (Figure 8a), probably because of the higher concentration of hydroxide anions in the 5 M KOH solution [51,52,53]. In addition, a stability test was conducted for 100 h with the same electrode, and it maintained its performance and stability even in the extreme environment (Figure 8b). Furthermore, SEM analysis of the electrode surface after the stability testing revealed that the nanosheet structure was maintained (Figure 8c).

## 4. Conclusions

In this study, CCO electrodes were synthesized by a practical and convenient method: electrodeposition and heat treatment. The low electrical conductivity of Co_3_O_4_, a non-noble metal electrode, was improved by the addition of copper. The effect of the changes in morphology and structure with respect to heat treatment temperature on the CCO electrode activities was investigated, allowing the optimization of the catalysts. The structure, chemical state, and morphology were analyzed using various techniques such as SEM, TEM, XRD and XPS. We confirmed that 250 °C annealed CCOH had the lowest overpotential of 276 mV at a current density of 20 mA/cm^2^ because all hydroxides are converted to oxides. In terms of the morphology, SEM analysis revealed that the surface maintained the nanosheet structure. Stability tests of the electrodes were conducted at 1 KOH. The synthesized electrodes showed high stability for 100 h at a current density of 20 mA/cm^2^. After the stability tests, the OER activity of the electrode was maintained at the same potential. The oxidation state of Co near surface through the OER increased, but the morphology and structural changes of electrode did not occur. The results indicate that the CCO electrodes are suitable and stable OER electrodes for use under extreme environments. Specifically, when stability tests were conducted at a high pH of 5 M KOH, high performance and stability were maintained and the nanosheet structure remained intact. Thus, the optimum CCO electrode was obtained by electrodeposition followed by heat treatment at 250 °C. 

## Figures and Tables

**Table 1:** Comparison of the reported OER activity.

Sample	Temperature	Electrolyte	Current Density (mA/cm^2^)	Overpotential (mV)	Ref.
CCO	Room temp.	1 M KOH	10	240	Our work
NiFe-OH-F				243	[38]
Fe-Co-P				252	[39]
NiFe-UMNs				260	[40]
1% Ce-NiFe-LDH/CNT				270	[41]
NiFe-LDH/CNT				299	[41]
CuO NF@G/CF				320	[42]
Co-N-C				321	[43]
Ni(OH)_2_				330	[44]
Co_3_O_4_				340	[45]
Ni-NDC/PANI-NF				361	[46]
Ni				365	[44]

## Data Availability

Data is available on the request from the corresponding author.
